# Relationship Between Cognitive and Clinical Insight at Different Durations of Untreated Attenuated Psychotic Symptoms in High-Risk Individuals

**DOI:** 10.3389/fpsyt.2021.753130

**Published:** 2021-11-10

**Authors:** LiHua Xu, Mei Zhang, ShuQin Wang, YanYan Wei, HuiRu Cui, ZhenYing Qian, YingChan Wang, XiaoChen Tang, YeGang Hu, YingYing Tang, TianHong Zhang, JiJun Wang

**Affiliations:** ^1^Shanghai Key Laboratory of Psychotic Disorders, Shanghai Mental Health Center, Shanghai Jiaotong University School of Medicine, Shanghai, China; ^2^Department of Nursing and Midwifery, Jiangsu College of Nursing, Huai'an, China; ^3^Department of Chinese Language Teaching, Shanghong Middle School, Shanghai, China; ^4^CAS Center for Excellence in Brain Science and Intelligence Technology (CEBSIT), Chinese Academy of Science, Shanghai, China; ^5^Institute of Psychology and Behavioral Science, Shanghai Jiao Tong University, Shanghai, China

**Keywords:** cognitive insight, clinical insight, clinical high risk for psychosis, schizophrenia spectrum disorders, duration of untreated attenuated psychotic symptoms

## Abstract

**Background:** This study examines whether cognitive insight is impaired in high-risk individuals with attenuated psychotic symptoms (APS) and explores the relationship between cognitive and clinical insight at different durations of untreated attenuated psychotic symptoms (DUAPS).

**Methods:** The Structured Interview for Psychosis high-risk Syndrome (SIPS) was used to identify APS individuals. APS (*n* = 121) and healthy control (HC, *n* = 87) subjects were asked to complete the Beck Cognitive Insight Scale (BCIS). Clinical insight of APS individuals was evaluated using the Schedule for Assessment of Insight (SAI). APS individuals were classified into four subgroups based on DUAPS, including 0–3, 4–6, 7–12, and >12 months. Power analysis for significant correlation was conducted using the WebPower package in R.

**Results:** Compared with HC subjects, APS individuals showed poorer cognitive insight, with lower scores on BCIS self-reflectiveness and composite index (BCIS self-reflectiveness minus BCIS self-certainty). Only when DUAPS was longer than 12 months did the significant positive correlation between cognitive and clinical insight obtain the power about 0.8, including the associations between self-reflectiveness and awareness of illness, self-reflectiveness and the total clinical insight, and composite index and awareness of illness. The positive associations of composite index with awareness of illness within 0–3 months DUAPS and with the total score of SAI when DUAPS > 12 months were significant but failed to obtain satisfactory power.

**Conclusions:** APS individuals may have impaired cognitive insight, demonstrating lower self-reflectiveness. The correlation between cognitive and clinical insight is associated with the duration of untreated attenuated psychotic symptoms.

## Introduction

Insight is a multi-dimensional concept (1). Insight into an illness or clinical insight includes awareness into mental illness, recognition of specific symptoms or relabeling, and acceptance of the need for treatment (2). Meanwhile, a metacognitive conceptualization of insight or cognitive insight includes self-reflectiveness and self-certainty (3). Self-reflectiveness refers to an individual's capacity and willingness to observe their mental processes and to consider alternative explanations; Self-certainty refers to individuals' overconfidence in the validity of their beliefs. Cognitive insight can be assessed using the Beck Cognitive Insight Scale (BCIS), with a high score on BCIS self-reflectiveness or a low score on BCIS self-certainty indicating good cognitive insight, which demonstrated sufficient convergent validity with the Scale to Assess Unawareness of Mental Disorder (SUMD) (3, 4). Cognitive insight does not involve judgment about psychiatric challenges but includes awareness of alterations in thought processes and reasoning styles, which differs from clinical insight (5) and has a complementary relationship with clinical insight (6, 7). In addition, cognitive insight is possibly more proximal to neurobiological vulnerabilities than clinical insight (6). It has been proposed that cognitive insight may underlie the development of adequate clinical insight (6).

However, the relationship between cognitive and clinical insight has been reported in the literature, with different conclusions. Most studies reported a close relationship between the two constructs (3, 7–13). In these studies, clinical insight was not always measured using the same tool. Initially, Beck et al. (3) estimated convergent validities of the BCIS subscales and composite index using SUMD, which measures clinical insight of individuals with psychotic disorders (4). Several subsequent studies have also found a relationship between cognitive and clinical insight measured using SUMD (7, 8, 12, 13). Other researchers have measured clinical insight using different versions of the Schedule for the Assessment of Insight (SAI) and have also found a relationship between cognitive and clinical insight (10, 11). Additionally, several studies using other scales that measure clinical insight, such as the Positive and Negative Syndrome Scale (14) and the Birchwood Insight Scale (9), have also yielded positive results. The relationship between the two constructs is mainly evident in an association of self-reflectiveness with awareness of illness and relabeling of symptoms (9, 13) independent of concurrent psychiatric symptoms and neurocognitive deficits (8). However, a few studies have failed to reveal a significant correlation between cognitive and clinical insight (15, 16).

Although numerous studies have focused on the relationship between cognitive and clinical insight, most of them involved subjects with first-episode or chronic schizophrenia. Clinical high risk for psychosis (CHR) is a proposed concept based on the prodromal period before the onset of psychosis (17). The conversion rate of CHR to psychosis within 2 years is ~ 30% (18, 19), and one criterion for evaluating the conversion outcome is whether the CHR individuals have completely lost their reality-testing ability, that is, whether they can judge—by testing reality—that their abnormal experience is due to their own psychological or mental problems rather than a real event (20). Previous research examined individuals at clinical high-risk state and speculated that the impairment of insight might exist before the onset of schizophrenia (21). Our previous study also found that the less the CHR individuals were aware of the unreality of their symptoms, the higher the probability that they would convert to psychosis (22). A recent meta-analysis included five studies on cognitive insight of CHR individuals and healthy control (HC) subjects and also revealed significant differences between the two groups in self-certainty, but no significant differences in self-reflectiveness and composite index (23). However, to our knowledge, no study has reported whether impaired clinical insight is related to cognitive insight during a high-risk period.

The duration of untreated attenuated psychotic symptoms (DUAPS) is another important concept (24–28). Studies have reported that longer DUAPS was correlated with reduced improvement in 12-month functional outcomes (24, 27). Our study investigated the rate of transition into psychosis in attenuated psychosis syndrome (APS, one syndrome of CHR) individuals with different DUAPS, and revealed higher conversion rates in the group with a DUAPS of 5–6 months than in those with DUAPS of 1–2, 3–4, and >6 months (26). In a previous study, we discussed that factors related to longer DUAPS may contribute to a lower risk of conversion to psychosis (26), and decided to explore whether there is also a regulatory effect of protective factors—such as self-reflectiveness—in individuals with longer DUAPS, which may be beneficial to improving the APS individuals' awareness into their mental illness. Therefore, the aims of this study were: 1) to examine whether the cognitive insight of APS individuals is impaired in comparison to HC subjects; 2) to analyze the relationship between cognitive and clinical insight in APS individuals with different DUAPS.

## Methods

### Procedure

This study was conducted according to the tenets of the Declaration of Helsinki. Participants were recruited from February 2019 to May 2021, and all of them signed written informed consent forms. For those younger than 18 years, both the participant and their next of kin or legal guardian provided informed consent. The inclusion and exclusion criteria were identical to those used in our previous study (29, 30). APS individuals, who sought help for the first time, were referred to the Shanghai at Risk for Psychosis-extended (SHARP-extended) program by outpatient physicians at the Shanghai Mental Health Center or its affiliate, the Shanghai Psychotherapy and Psychological Counseling Center, and were interviewed by trained raters using the Chinese version of the Structured Interview for Psychosis high risk Syndrome (SIPS). HC subjects with no history of psychiatric disorders were recruited from a junior high school (*n* = 20) and a senior high school (*n* = 31) in Anhui province, a college in Jiangsu province (*n* = 21), and a junior high school in Shanghai (*n* = 15).

### Participants

In this study, 121 APS and 87 HC subjects were included, all of whom completed a self-report on the BCIS. Demographic information and BCIS scores are presented in Table 1. The APS and HC groups matched in terms of demographic variables. APS individuals were further classified into four subgroups according to their DUAPS: 0–3 months (short DUAPS, SDUAPS), 4–6 months (*medium* DUAPS, MDUAPS), 7–12 months (*long* DUAPS, LDUAPS), and >12 months (*superlong* DUAPS, SLDUAPS). The numbers (percentages of total *N*) of individuals with SDUAPS, MDUAPS, LDUAPS, and SLDUAPS were 39 (32.23%), 30 (24.79%), 24 (19.83%), and 28 (23.14%), respectively. DUAPS was defined as the period between the onset of attenuated psychotic symptoms and the commencement of professional help at mental health services (25–27). It was measured retrospectively based on the information from the SIPS interview (25), and the Kendall's coefficient of concordance among four raters (Zhang T., Xu L., Wei Y., and Wang Y.) was 0.82.

**Table 1:** Comparison of demographic and clinical characteristics.

	**APS**	**HC**	**t/χ ^**2**^**	***p*-value**
	**(*N* = 121)**	**(*N* = 87)**		
Age	18.37 ± 5.59	18.24 ± 3.56	0.206	0.837
Gender (male/female)	54/67	34/53	0.638	0.424
Education (year)	10.61 ± 3.52	10.75 ± 2.52	0.324	0.746
Self-reflectiveness	14.41 ± 3.82	21.28 ± 3.49	13.26	<0.001
Self-certainty	9.83 ± 2.90	14.71 ± 2.07	14.18	<0.001
Composite index	4.59 ± 4.84	6.56 ± 3.33	3.49	0.001
SAI-illness	4.45 ± 1.29	—	—	—
SAI-symptoms	2.60 ± 0.91	—	—	—
SAI-treatment	3.07 ± 1.01	—	—	—
SAI-total	10.11 ± 2.80	—	—	—
SOPS-P	10.74 ± 3.60	—	—	—
SOPS-N	13.63 ± 5.56	—	—	—
SOPS-D	6.41 ± 2.98	—	—	—
SOPS-G	10.38 ± 3.03	—	—	—
DUAPS	8.13 ± 8.79	—	—	—
SDUAPS (0–3 months) (num/percent)	39 (32.23%)	—	—	—
MDUAPS (4–6 months) (num/percent)	30 (24.79%)	—	—	—
LDUAPS (7–12 months) (num/percent)	24 (19.83%)	—	—	—
SLDUAPS (>12 months) (num/percent)	28 (23.14%)	—	—	—

*APS refers to individuals with attenuated psychotic symptoms at clinical high-risk for psychosis; HC healthy controls; SAI is short for the schedule of assessment of insight; SAI-illness refers to the dimension of awareness into illness; SAI-symptoms refers to relabeling of specific symptoms; SAI-treatment refers to treatment compliance; SAI-total refers to the total score of SAI; SOPS-P is the total score of positive symptoms of the Scale of Psychotic-risk Syndromes (SOPS); SOPS-N is the total score of negative symptoms; SOPS-D is the total score of disorganized symptoms; SOPS-G is the total score of general symptoms; DUAPS refers to the duration from the onset of prodromal symptoms to the first visit to seek professional help; SDUAPS is short DUAPS (0–3 months); MDUAPS is medium (4–6 months); LDUAPS is long (7–12 months); SLDUAPS is superlong (>12 months)*.

### Measures

#### Structured Interview for Psychosis High-Risk Syndrome

The most important aspect of SIPS is the Scale of Psychosis-risk Syndromes (SOPS) (31, 32), which contains four dimensions: positive, negative, disorganized, and general symptoms. The Global Assessment of Functioning (GAF), criteria for schizotypal personality, and family history of mental illness are included in the structured interview. According to the Criteria of Psychosis-risk Syndromes (COPS), one or more of three psychosis high-risk syndromes, such as APS, brief intermittent psychotic syndrome, and genetic risk and deterioration syndrome (GRDS), is determined. This study only included APS individuals with or without GRDS, considering that the DUAPS in this study is primarily concerned with attenuated psychotic symptoms. Our team translated the SIPS into Chinese and established its reliability and validity (18, 19, 33).

#### The Beck Cognitive Insight Scale

The BCIS is a self-report questionnaire with 15 items and two subscales, including self-reflectiveness and self-certainty (3). Self-reflectiveness, measured by nine items, refers to the ability to evaluate mental symptoms and consider alternative explanations for the symptoms. Self-certainty, measured by six items, refers to the subjects' overconfidence in their explanation of anomalous experiences and the intensity of resistance to changing their beliefs. Higher scores on self-reflectiveness or lower scores on self-certainty indicate better cognitive insight. The composite index is calculated from the score of self-reflectiveness minus that of self-certainty, indicating adjusted self-reflectiveness. Participants were asked to rate on a four-point scale from “strongly disagree” to “strongly agree” with the statement for each item. The BCIS has good reliability and validity (3, 34).

#### Schedule of Assessment of Insight

David, (2) proposed that clinical insight contains at least three dimensions, including: awareness of the illness, the capacity to relabel psychotic experiences as abnormal, and treatment compliance. The SAI was developed according to each facet of insight and has been proven to be applicable to APS individuals (21). SAI is a semi-structured interview tool that includes seven items, with a total score of 14 (0–6 for awareness of illness, 0–4 for relabeling of specific symptoms, and 0–4 for treatment compliance). The SAI has recognized reliability and validity (35).

### Statistical Analysis

SPSS version 26.0 (SPSS Inc., Chicago, IL, USA) was used for the analysis. An independent-samples *t*-test was used to compare continuous variables between APS and HC subjects. A chi-squared test was conducted to compare the differences in the proportions of categorical variables. After APS individuals were divided into four subgroups, the sample sizes of two subgroups were <30, and some variables were non-normally distributed; therefore, the Kruskal-Wallis test was conducted to compare the differences in each variable among the APS subgroups with different DUAPS. Spearman correlation analysis was conducted to explore the associations of cognitive insight with clinical symptoms and clinical insight in each APS subgroup. Considering the small sample size of each group, we used the WebPower package (36) in *R* to conduct post hoc power analysis for the correlation results. The level of significance was set at 0.05 (two-tailed).

## Results

### Descriptive

No significant differences in age, gender distribution, and years of education between the APS and HC (*p* > 0.05) groups were found. The independent samples *t*-test revealed that APS individuals had significantly lower scores than HC subjects (*p* < 0.05) on self-reflectiveness, self-certainty, and the composite index. The results and clinical information of APS individuals are shown in Table 1.

### Comparison of Different DUAPS Subgroups

The Kruskal-Wallis test was conducted to compare the differences in each variable among the different DUAPS subgroups. The results showed no significant differences among the subgroups in terms of age, gender distribution, years of education, cognitive and clinical insight, and the total score of each dimension of SOPS (*p* > 0.05) (see Table 2).

**Table 2:** Demographic, cognitive and clinical insight, and other clinical variables of APS subgroups with different DUAPS.

**Items**	**SDUAPS**	**MDUAPS**	**LDUAPS**	**SLDUAPS**	**Z/χ ^**2**^**	***p-*value**
	**(*n* = 39)**	**(*n* = 30)**	**(*n* = 24)**	**(*n* = 28)**		
Age	17.00 (7.00)	16.50 (6.00)	15.00 (6.00)	17.00 (9.00)	2.625	0.453
Gender (male/female)	18/21	14/16	7/17	15/13	3.315	0.346
Education (year)	10.00 (5.00)	9.50 (3.00)	9.00 (5.00)	10.50 (7.00)	2.497	0.476
Self-reflectiveness	15.00 (4.00)	15.00 (6.25)	14.00 (4.00)	14.00 (8.00)	0.942	0.815
Self-certainty	10.00 (4.00)	10.00 (4.50)	9.50 (4.75)	10.00 (3.00)	2.089	0.554
Composite index	5.00 (5.00)	5.00 (8.25)	4.00 (5.75)	6.50 (8.75)	0.382	0.944
SAI-treatment	3.00 (2.00)	3.00 (2.00)	4.00 (1.00)	3.00 (1.00)	2.927	0.403
SAI-illness	5.00 (1.00)	5.00 (1.25)	5.00 (1.00)	4.50 (2.75)	0.190	0.979
SAI-symptoms	3.00 (1.00)	2.50 (1.00)	3.00 (0.75)	2.00 (1.00)	2.250	0.522
SAI-total	11.00 (3.00)	10.00 (4.25)	11.00 (2.75)	10.00 (4.75)	0.934	0.817
SOPS-P	11.00 (5.00)	9.50 (7.25)	10.50 (4.75)	12.00 (4.75)	1.962	0.580
SOPS-N	13.00 (7.00)	13.50 (7.50)	12.00 (6.75)	13.00 (7.50)	0.605	0.895
SOPS-D	6.00 (5.00)	5.50 (4.25)	6.00 (4.50)	7.00 (4.00)	5.055	0.168
SOPS-G	10.00 (3.00)	11.00 (4.25)	10.50 (5.75)	11.00 (4.00)	0.151	0.985

*The value in the table for each continuous variable is the median (quartile deviation)*.

### Correlation Analysis Among Different DUAPS Subgroups

#### Cognitive Insight and SOPS Ratings

**Figure 1:**
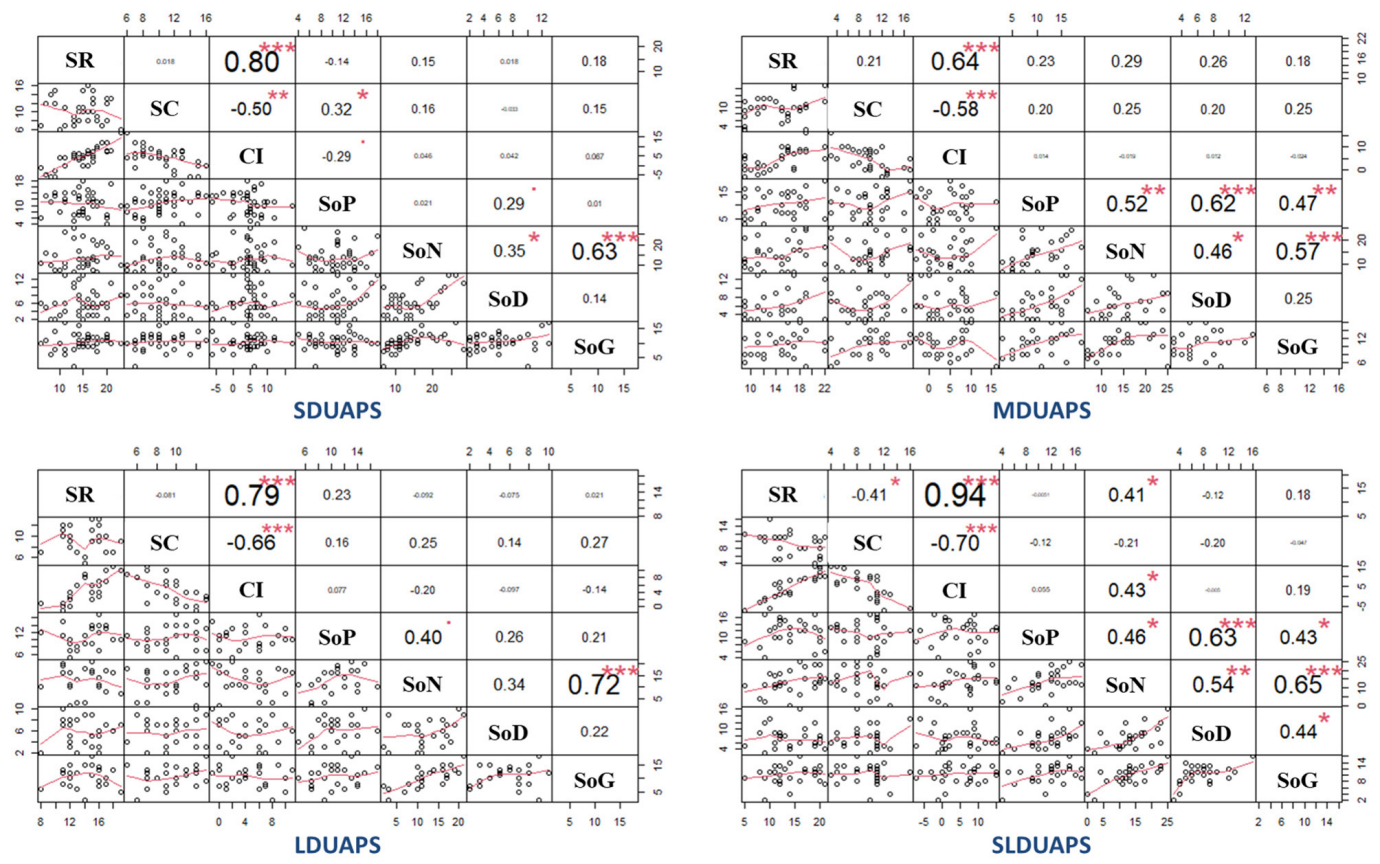
Correlation (Spearman) between cognitive insight and clinical symptoms. DUAPS is the duration from the onset of prodromal symptoms to the first visit to seek professional help; SDUAPS, MDUAPS, LDUAPS, and SLDUAPS refer to 0–3, 4–6, 7–12, and >12 months, respectively; SR refers to self-reflectiveness; SC refers to self-certainty; CI refers to composite index; SoP refers to the total score of positive symptoms of the Scale of Psychotic-risk Syndromes (SOPS); SoN refers to the total score of negative symptoms of SOPS; SoD refers to the total score of disorganized symptoms; SoG refers to the total score of general symptoms. ^*^p < 0.05; **p < 0.01; ***p < 0.001.

To explore the relationship between cognitive insight and the total score of each symptom dimension of SOPS from short (0–3 months) to super long (>12 months) DUAPS, Spearman correlation analysis was conducted. The results showed that self-certainty was positively correlated with the total score of positive symptoms (*p* < 0.05, power value = 0.52) in the first 3 months after the onset of prodromal symptoms. Self-reflectiveness and the composite index demonstrated a positive correlation with the total score of negative symptoms (*p* < 0.05) when DUAPS was over 12 months, and the power values were 0.60 and 0.65, respectively (see Figure 1).

#### Cognitive and Clinical Insight

**Figure 2:**
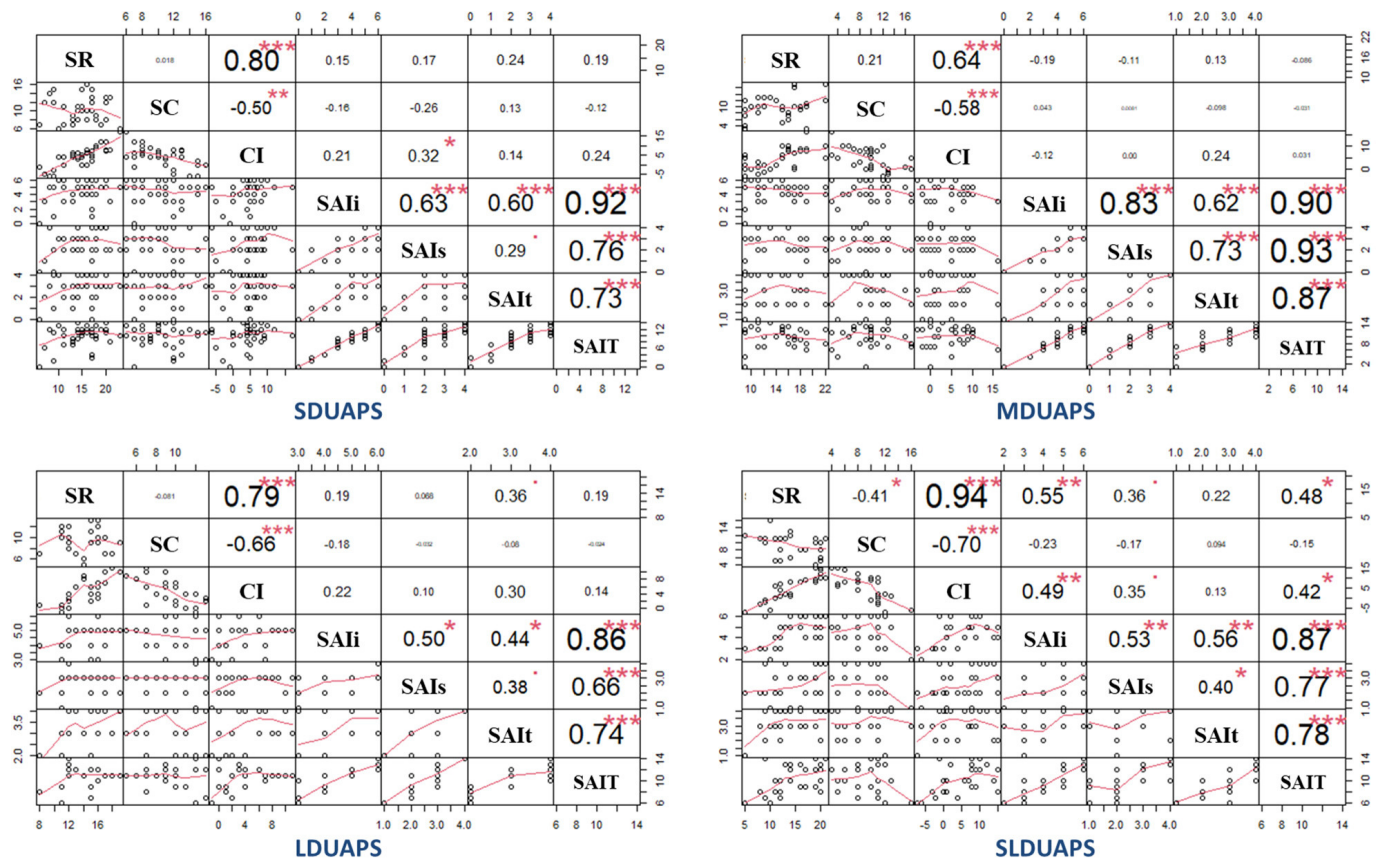
Correlation (Spearman) between cognitive and clinical insight. DUAPS is the duration from the onset of prodromal symptoms to the first visit to seek professional help; SDUAPS, MDUAPS, LDUAPS, and SLDUAPS refer to 0–3, 4–6, 7–12, and >12 months, respectively; SR refers to self-reflectiveness; SC refers to self-certainty; CI refers to composite index; SAI is short for the schedule of assessment of insight; SAIi refers to the dimension of awareness into illness; SAIs refers to relabeling of specific symptoms; SAIt refers to treatment compliance; SAIT refers to the total score of SAI; *p < 0.05; **p < 0.01; ***p < 0.001.

Spearman correlation analysis was also conducted to explore the relationship between cognitive and clinical insight. The results showed that within the first 3 months of DUAPS, the composite index demonstrated a positive correlation with SAI symptoms (*p* < 0.05, power value = 0.52), and when DUAPS was over 12 months, self-reflectiveness and composite index positively correlated with SAI-illness (*p* < 0.05, power value: 0.88 and 0.78) and with SAI-Total significantly (*p* < 0.05, power value: 0.76 and 0.63) (see Figure 2).

## Discussion

To our knowledge, no studies have analyzed the relationship between cognitive and clinical insight at different DUAPS. This study found, compared to HC subjects, APS individuals had impaired cognitive insight in terms of lower scores on self-reflectiveness and composite index of BCIS, and further revealed that BCIS self-reflectiveness and BCIS composite index were associated with clinical insight measured by SAI, especially in the SLDUAPS group. No significant correlations were found between BCIS self-certainty and clinical insight in each group.

The difference in cognitive insight between CHR and HC subjects was inconclusive in previous studies (37–40). Dondé et al. (23) conducted a meta-analysis; they found that CHR individuals had poorer cognitive insight in terms of higher scores on self-certainty than HC subjects, but failed to reveal significant differences in self-reflectiveness and composite index. However, in the present study, APS individuals scored lower than HC subjects not only in self-reflectiveness but also in self-certainty. The higher scores on self-reflectiveness and self-certainty of HC subjects may be related to the setting of this study. In the school, self-report questionnaires were handed out to students by their teachers; therefore, it is possible that students wanted to perform better. For them, self-certainty may reflect affirmation and confidence of their own experiences. However, this study unified the guidelines and set up an anonymous questionnaire; thus, we speculate that this effect is small. Alternatively, Chinese culture may be a factor. Wang and Ren, found that Chinese students rated themselves significantly higher than American students did when only measuring facts or the status of self-related items without involving self-evaluation (41). Additionally, Van Camp et al. (42) proposed that the current form of cognitive insight was less valid for individuals without psychosis, because the BCIS emphasizes unusual experiences. However, several studies have observed cognitive insight of HC subjects using the BCIS and conducted analysis (23, 37, 38, 40). Overall, this study indicates that APS individuals may have shortcomings in self-reflectiveness, but the same cannot be concluded with respect to self-certainty. The results of this study may only be representative of the APS individuals who came to seek help. Non-help seekers among APS individuals may function well in society and maintain good cognitive insight.

Our study compared the scores of the four APS subgroups on cognitive insight, clinical insight, and symptomatology, but failed to find differences among the four subgroups with different DUAPS. However, the associations of cognitive insight with clinical insight and symptomatology demonstrated varying patterns in different subgroups. The results revealed that self-certainty positively correlated with the total score of positive symptoms of SOPS, while self-reflectiveness and composite index positively correlated with the total score of negative symptoms, which is consistent with previous studies. Previous studies have reported that cognitive insight was closely associated with psychological symptomatology (38, 43–45). Self-certainty was reported to be associated with delusional beliefs (38, 45), while self-reflectiveness was reported to be associated with depressive symptomatology (43, 46). However, this study may only suggest potential correlations between cognitive insight and clinical symptoms, because the power values were relatively low (0.52–0.65). The results may be related to the small sample size.

This study revealed that self-certainty was positively correlated with positive symptoms at the earliest period (0–3 months) after the onset of high-risk symptoms, but not at later periods. We propose that during the initial period, the higher self-certainty APS individuals have, the more obvious the symptoms they report; and the more severe the positive symptoms are, the more convinced the patients are of the symptoms. However, because APS individuals still have partial or intact reality-testing ability (20, 22, 47) and realize the unreality of the symptoms in a later period, the correlation between the degree of self-certainty and the severity of positive symptoms possibly weakened or disappeared. Moreover, the results on the relationships between self-reflectiveness/composite index and negative symptoms are consistent with previous studies. Several studies have reported that self-reflectiveness was closely associated with negative affect, such as depression and anxiety (43, 46, 48–51), which have strong associations with negative symptoms (52–54). In addition, clinical insight, which is closely related to self-reflectiveness, has also been reported to be positively associated with depression (55) and negative symptoms (56). This study further revealed that positive correlations between self-reflectiveness/composite index and negative symptoms existed in the APS subgroup with DUAPS longer than 12 months, but not in the other APS subgroups. It is inferred that self-reflectiveness/composite index may have an indirect effect on negative symptoms through clinical insight or negative affect; thus, the potential correlations was observed at a later period.

In terms of the relationship between cognitive and clinical insight, this study failed to reveal the correlations of self-certainty with any dimension of clinical insight (*p* > 0.05). This result is inconsistent with previous studies, which have found significant correlations (9) or trend-level correlations (12, 16) between self-certainty and clinical insight. Although Beck et al. (3) did not find significant correlations between self-certainty and the SUMD items, the correlation coefficient suggested moderate effect sizes, considering that the small sample size affected the significance of the magnitudes of the correlations. Given the results of this study, we propose that there may be regulatory factors between self-certainty and clinical insight, such as reality testing ability. Even when individuals are characterized by a high level of self-certainty or an increased self-certainty level due to abnormal experiences, when they update their original ideas by testing reality, clinical insight is likely to be protected from the influence of self-certainty.

The results regarding the positive association of self-reflectiveness and composite index with clinical insight are consistent with previous studies (3, 7, 9, 12). The composite index was calculated by subtracting the self-certainty score from the self-reflectiveness score, and it was considered to be self-reflectiveness adjusted for self-certainty (3). However, the correlation between composite index and relabeling of symptoms (SAI-symptoms) at the earliest period (DUAPS of 0–3 months) failed to obtain a power of 0.80 (actually 0.52). The remarkable results about the correlations of self-reflectiveness and composite index with awareness of illness and the total score of clinical insight mainly occurred in the APS subgroup with DUAPS > 12 months. The power values were acceptable (0.76–0.88), except for the correlation between composite index and the total score of clinical insight (0.63). It is indicated that at the beginning of the onset of abnormal experiences, APS individuals' recognition of symptoms may be affected by self-reflectiveness, and when abnormal experiences persist for a long time (more than 1 year), self-reflectiveness may influence APS individuals' awareness of mental illness.

The novelty of this study is that the relationships of cognitive insight with clinical insight were analyzed at different periods after the onset of abnormal experiences in high-risk individuals. However, this study has some limitations. First, the data of HC subjects in this study were collected by teachers in schools and were not implemented in the same hospital environment as that of the APS individuals, which may have affected the results. Second, according to the results of this study, APS individuals may have shortcomings in self-reflectiveness, but whether a self-certainty anomaly exists is a topic that requires further analysis, which can be accomplished by recruiting patients with psychosis. Third, because the sample size of each APS group with different DUAPS was small, the results need to be further verified by expanding the sample size.

## Conclusion

This study found inconsistent results when comparing the differences in cognitive insight using the BCIS between APS and HC subjects. APS individuals may have self-reflectiveness shortcomings, but whether their self-certainty level is abnormal requires further research. Moreover, self-certainty may have different meanings for APS and HC subjects. Our study further divided APS individuals into four subgroups according to the DUAPS and analyzed the associations of cognitive insight with clinical symptoms and clinical insight. The results indicate that cognitive insight may have an impact on clinical insight when the attenuated positive symptoms appeared at the beginning (0–3 months) or lasted for a longer time (>6 months). At the beginning of the onset of attenuated positive symptoms, self-certainty demonstrated potential association with the severity of positive symptoms, while self-reflectiveness may enhance the ability of APS individuals to relabel attenuated symptoms. The results suggest that after positive symptoms have lasted for 12 months, APS individuals with higher self-reflectiveness may have better clinical insight, but simultaneously, may experience significant negative symptoms.

## Data Availability Statement

The raw data supporting the conclusions of this article will be made available by the authors, without undue reservation.

## Ethics Statement

The studies involving human participants were reviewed and approved by Research Ethics Committee of the Shanghai Mental Health Center. Written informed consent to participate in this study was provided by the participants' legal guardian/next of kin.

## Author Contributions

LX: wrote the first draft of manuscript and conducted the statistical analyses. MZ and SW: recruited healthy controls. YYW, HC, ZQ, and YCW: interviewed participants and organized the primary data. XT, YH, and YT: reviewed and revised the draft. TZ and JW: designed the study and provided supervision in the implementation of the study. All authors have approved the final manuscript.

## Funding

This study was supported by Ministry of Science and Technology of China, National Key R&D Program of China (2016YFC1306800); National Natural Science Foundation of China (81671332, 81971251, 81671329, 81871050, and 81901832); Shanghai Municipal Science and Technology Major Project (2018SHZDZX01) and ZJLab; Science and Technology Commission of Shanghai Municipality (16JC1420200, 16ZR1430500, 19410710800, 19411950800, 19411969100, 19441907800, 19ZR1477800, and 20ZR1448600); Shanghai Municipal Health Commission (202040361); Project of the Key Discipline Construction, Shanghai 3-Year Public Health Action Plan (GWV-10.1-XK18); Shanghai Clinical Research Center for Mental Health (19MC1911100); Clinical Research Plan of SHDC (SHDC2020CR4066); Clinical Research Center at Shanghai Jiao Tong University School of Medicine (DLY201817); Shanghai Jiao Tong University Foundation (ZH2018ZDB03, ZH2018QNB19), The Clinical Research Center at Shanghai Mental Health Center (CRC2018ZD01, CRC2018ZD04, CRC2018YB01, and CRC2019ZD02), Shanghai Mental Health Center (2019-zd01).

## Conflict of Interest

The authors declare that the research was conducted in the absence of any commercial or financial relationships that could be construed as a potential conflict of interest.

## Publisher's Note

All claims expressed in this article are solely those of the authors and do not necessarily represent those of their affiliated organizations, or those of the publisher, the editors and the reviewers. Any product that may be evaluated in this article, or claim that may be made by its manufacturer, is not guaranteed or endorsed by the publisher.
