# Corrigendum: Relationship Between Cognitive and Clinical Insight at Different Durations of Untreated Attenuated Psychotic Symptoms in High-Risk Individuals

**DOI:** 10.3389/fpsyt.2022.839315

**Published:** 2022-02-08

**Authors:** LiHua Xu, Mei Zhang, ShuQin Wang, YanYan Wei, HuiRu Cui, ZhenYing Qian, YingChan Wang, XiaoChen Tang, YeGang Hu, YingYing Tang, TianHong Zhang, JiJun Wang

**Affiliations:** ^1^Shanghai Key Laboratory of Psychotic Disorders, Shanghai Mental Health Center, Shanghai Jiaotong University School of Medicine, Shanghai, China; ^2^Department of Nursing and Midwifery, Jiangsu College of Nursing, Huai'an, China; ^3^Department of Chinese Language Teaching, Shanghong Middle School, Shanghai, China; ^4^CAS Center for Excellence in Brain Science and Intelligence Technology (CEBSIT), Chinese Academy of Science, Shanghai, China; ^5^Institute of Psychology and Behavioral Science, Shanghai Jiao Tong University, Shanghai, China

**Keywords:** cognitive insight, clinical insight, clinical high risk for psychosis, schizophrenia spectrum disorders, duration of untreated attenuated psychotic symptoms

There is an error in the title of the original article. The **Title** was incorrectly given as ‘Relationship Between Cognitive and Clinical Insight at Different Durations of Untreated Attentional Psychotic Symptoms in High-Risk Individuals.' The correct **Title** is ‘Relationship Between Cognitive and Clinical Insight at Different Durations of Untreated Attenuated Psychotic Symptoms in High-Risk Individuals.'

There is an error in the **Funding** statement. ‘Shanghai Municipal Science and Technology Major Project (2018SHZDZX01) and ZJLab' was written twice.

An author name was incorrectly spelled as ‘ShuQing Wang'. The correct spelling is ‘ShuQin Wang'.

In the original article, the reference for ‘Sagayadevan V, Jeyagurunathan A, Lau YW, Shafie S, Chang S, Ong HL, et al. Cognitive insight and quality of life among psychiatric outpatients. BMC Psychiatry. (2019) 19:201. doi: 10.1186/s12888-019-2163-y' was incorrectly cited. It should be deleted.

The reference for citation 35, ‘Xu Z, Guo Z, Fu Z, Wang N, Zhang Y. Reliability and validity of the Chinese version of the schidule for assessment of insight (in Chinese). Chinese J Behav Med Brain Sci. (2013) 22:752–4. doi: 10.3760/cma.j.issn.1674-6554.2013.08.024' was incorrectly written. It should be ‘35. Xu Z, Guo Z, Fu Z, Wang N, Zhang Y. Reliability and validity of the Chinese version of the schedule for assessment of insight (in Chinese). Chinese J Behav Med Brain Sci. (2013) 22:752–4. doi: 10.3760/cma.j.issn.1674-6554.2013.08.024.'

In the original article, there was an error. The original article stated that “Sagayadevan et al. (55) also reported that psychiatric outpatients with higher self-reflectiveness scored higher on the environment domain but lower on the social relationships domain of quality of life.”, but this reference reported that higher self-certainty, not lower self-reflectiveness, was associated with lower on the social relationships domain of quality of life.

A correction has therefore been made to the **Discussion, fourth paragraph:**

‘This study revealed that self-certainty was positively correlated with positive symptoms at the earliest period (0–3 months) after the onset of high-risk symptoms, but not at later periods. We propose that during the initial period, the higher self-certainty APS individuals have, the more obvious the symptoms they report; and the more severe the positive symptoms are, the more convinced the patients are of the symptoms. However, because APS individuals still have partial or intact reality-testing ability (20, 22, 47) and realize the unreality of the symptoms in a later period, the correlation between the degree of self-certainty and the severity of positive symptoms possibly weakened or disappeared. Moreover, the results on the relationships between self-reflectiveness/composite index and negative symptoms are consistent with previous studies. Several studies have reported that self-reflectiveness was closely associated with negative affect, such as depression and anxiety (43, 46, 48–51), which have strong associations with negative symptoms (52–54). In addition, clinical insight, which is closely related to self-reflectiveness, has also been reported to be positively associated with depression (55) and negative symptoms (56). This study further revealed that positive correlations between self-reflectiveness/composite index and negative symptoms existed in the APS subgroup with DUAPS longer than 12 months, but not in the other APS subgroups. It is inferred that self-reflectiveness/composite index may have an indirect effect on negative symptoms through clinical insight or negative affect; thus, the potential correlations was observed at a later period.'

The authors apologize for these errors and state that they do not change the scientific conclusions of the article in any way. The original article has been updated.

## Publisher's Note

All claims expressed in this article are solely those of the authors and do not necessarily represent those of their affiliated organizations, or those of the publisher, the editors and the reviewers. Any product that may be evaluated in this article, or claim that may be made by its manufacturer, is not guaranteed or endorsed by the publisher.

## References

[20] Solis M. Prevention: before the break. Nature. (2014) 508:S12–3. 10.1038/508S12a 24695328

[22] Xu L Zhang T Zheng L Li H Tang Y Luo X. Psychometric properties of prodromal questionnaire-brief version among Chinese help-seeking individuals. PLoS ONE. (2016) 11:e0148935. 10.1371/journal.pone.0148935 26859774 PMC4747512

[43] García-Mieres H De Jesús-Romero R Ochoa S Feixas G. Beyond the cognitive insight paradox: self-reflectivity moderates the relationship between depressive symptoms and general psychological distress in psychosis. Schizophr Res. (2020) 222:297–303. 10.1016/j.schres.2020.05.027 32518005

[46] Palmer EC Gilleen J David AS. The relationship between cognitive insight and depression in psychosis and schizophrenia: a review and meta-analysis. Schizophr Res. (2015) 166:261–8. 10.1016/j.schres.2015.05.032 26095015

[47] Shepherd A. Psychosis as a failure of reality testing. Br J Psychiatry. (2014) 204:242. 10.1192/bjp.204.3.242 24590979

[48] Liu J Chan TCT Chong SA Subramaniam M Mahendran R. Impact of emotion dysregulation and cognitive insight on psychotic and depressive symptoms during the early course of schizophrenia spectrum disorders. Early Interv Psychiatry. (2020) 14:691–7. 10.1111/eip.12895 31692276

[49] Konsztowicz S Lepage M. The role of illness engulfment in the association between insight and depressive symptomatology in schizophrenia. J Psychiatr Res. (2019) 111:1–7. 10.1016/j.jpsychires.2018.11.001 30658135

[50] Nakajima M Takano K Tanno Y. Adaptive functions of self-focused attention: insight and depressive and anxiety symptoms. Psychiatry Res. (2017) 249:275–80. 10.1016/j.psychres.2017.01.026 28135598

[51] Misdrahi D Denard S Swendsen J Jaussent I Courtet P. Depression in schizophrenia: the influence of the different dimensions of insight. Psychiatry Res. (2014) 216:12–6. 10.1016/j.psychres.2014.01.039 24529815

[52] Herniman SE Phillips LJ Wood SJ Cotton SM Liemburg EJ Allott KA. Interrelationships between depressive symptoms and positive and negative symptoms of recent onset schizophrenia spectrum disorders: a network analytical approach. J Psychiatr Res. (2021) 140:373–80. 10.1016/j.jpsychires.2021.05.038 34144441

[53] Quek YF Yang Z Dauwels J. Lee J. The impact of negative symptoms and neurocognition on functioning in MDD and schizophrenia. Front Psychiatry. (2021) 12:648108. 10.3389/fpsyt.2021.648108 34381384 PMC8350050

[54] Calderon-Mediavilla M Vila-Badia R Dolz M Butjosa A Barajas A Del Cacho N. Depressive symptoms and their relationship with negative and other psychotic symptoms in early onset psychosis. Eur Child Adolesc Psychiatry. (2021) 30:1383–90. 10.1007/s00787-020-01618-0 32865655

[55] Cobo J Labad J Pousa E Nieto L Ochoa S Usall J. Exploring the relationship of insight with psychopathology and gender in individuals with schizophrenia spectrum disorders with structural equation modelling. Arch Womens Ment Heal. (2020) 23:643–55. 10.1007/s00737-020-01031-1 32385644

[56] Ho E Lee M Lai C Hui M Pui K Chan K. The role of symptoms and insight in mediating cognition and functioning in fi rst episode psychosis. Schizophr Res. (2019) 206:251–6. 10.1016/j.schres.2018.11.009 30449592

